# Recognizing Frustration of Drivers From Face Video Recordings and Brain Activation Measurements With Functional Near-Infrared Spectroscopy

**DOI:** 10.3389/fnhum.2018.00327

**Published:** 2018-08-17

**Authors:** Klas Ihme, Anirudh Unni, Meng Zhang, Jochem W. Rieger, Meike Jipp

**Affiliations:** ^1^Department of Human Factors, Institute of Transportation Systems, German Aerospace Center (DLR), Braunschweig, Germany; ^2^Department of Psychology, University of Oldenburg, Oldenburg, Germany

**Keywords:** frustration, driver state recognition, facial expressions, functional near-infrared spectroscopy, adaptive automation

## Abstract

Experiencing frustration while driving can harm cognitive processing, result in aggressive behavior and hence negatively influence driving performance and traffic safety. Being able to automatically detect frustration would allow adaptive driver assistance and automation systems to adequately react to a driver’s frustration and mitigate potential negative consequences. To identify reliable and valid indicators of driver’s frustration, we conducted two driving simulator experiments. In the first experiment, we aimed to reveal facial expressions that indicate frustration in continuous video recordings of the driver’s face taken while driving highly realistic simulator scenarios in which frustrated or non-frustrated emotional states were experienced. An automated analysis of facial expressions combined with multivariate logistic regression classification revealed that frustrated time intervals can be discriminated from non-frustrated ones with accuracy of 62.0% (mean over 30 participants). A further analysis of the facial expressions revealed that frustrated drivers tend to activate muscles in the mouth region (chin raiser, lip pucker, lip pressor). In the second experiment, we measured cortical activation with almost whole-head functional near-infrared spectroscopy (fNIRS) while participants experienced frustrating and non-frustrating driving simulator scenarios. Multivariate logistic regression applied to the fNIRS measurements allowed us to discriminate between frustrated and non-frustrated driving intervals with higher accuracy of 78.1% (mean over 12 participants). Frustrated driving intervals were indicated by increased activation in the inferior frontal, putative premotor and occipito-temporal cortices. Our results show that facial and cortical markers of frustration can be informative for time resolved driver state identification in complex realistic driving situations. The markers derived here can potentially be used as an input for future adaptive driver assistance and automation systems that detect driver frustration and adaptively react to mitigate it.

## Introduction

Imagine driving through a city during rush hour on the way to an important meeting. You started a little late and realize that with the dense traffic conditions, it will be hard to arrive at the meeting in time. You are becoming increasingly annoyed by the driver in front of you who is driving provocatively slowly and causes unnecessary extra stops at traffic lights. In addition, the myriads of construction sites along your way further worsen the situation. After yet another red light, you are really frustrated and it appears unbearable to you to wait behind the bus right after the light turned green. You accelerate to overtake the bus but fail to see the pedestrian crossing the street and heading to the bus.

The above story is one example of how frustration can affect driving in a negative way and most readers have likely experienced one or the other situation. Frustration can be seen as an aversive emotional state resulting when goal-directed behavior is blocked (Lazarus, 1991) that is associated with negative valence and slightly elevated arousal (Russell, 1980; Scherer, 2005). As driving is a goal-directed behavior (e.g., reaching the destination in time in the above example), blocking events, as described above, can induce frustration and eventually lead to aggressive (driving) behavior (Ekman and Friesen, 2003; Lee, 2010; Jeon, 2015). In addition, frustration can have negative effects on cognitive processes important for driving such as attention, judgment and decision making (Jeon, 2015). Jeon (2012) suggested that negative emotions may have a worse influence on driving performance than distraction or secondary tasks, as drivers normally are aware of the secondary task and can pro-actively compensate for it. Conversely, negative emotions may degrade driving performance without attempts for compensation (Jeon, 2012). Taken together, reducing frustration during driving is an important step towards improving road safety (e.g., zero-vision of the European Commission, as expressed in the White Paper on Transportation, European Commission, 2011).

In order to reduce frustration and its potential negative consequences in future intelligent driver assistance systems by means of emotion-aware systems (e.g., Picard and Klein, 2002), it is necessary to automatically assess the drivers’ current level of frustration. One potential indicator of emotions is the momentary facial expression. Humans use facial expressions to communicate their emotions and these facial expressions appear to be relatively idiosyncratic to specific emotions (Ekman and Friesen, 2003; Erickson and Schulkin, 2003) and hence may discriminate between different emotions. Moreover, brain activations are the physiological basis of emotions, appraisal processes as well as subjective experiences (Scherer, 2005) and may allow to objectively discriminate frustrated from non-frustrated subjective states. Therefore, we will investigate whether facial expressions and brain activation patterns are indicative for frustration while driving.

Humans communicate emotions by changing the configuration of the activation of their facial muscles which is fundamental to understand each other in social interaction (Erickson and Schulkin, 2003). Following from this, the idea is to equip machines like vehicles with the same capability to read facial expressions in order to gain the ability to interpret the driver’s current emotional state and eventually become empathic (e.g., Bruce, 1992; Picard and Klein, 2002). This vision becomes realistic with the recent progress in the fields of image processing and machine learning making it possible to automatically track changes in facial activity from video recordings of the face (Bartlett et al., 2008; Hamm et al., 2011; Gao et al., 2014). Still, to the best of our knowledge, so far, only few studies investigated the facial features accompanying frustration and whether these can be used to discriminate frustration from other emotional states. For example, a study by Malta et al. (2011) used facial features to detect frustration in real world situations but did not report the discriminative features. Studies from human-computer interaction (HCI) linked frustration to increased facial muscle movement in the eye brow and mouth area (D’Mello et al., 2005; Grafsgaard et al., 2013). In addition, a recent study investigating facial activity of frustrated drivers found that muscles in the mouth region (e.g., tightening and pressing of lips) were more activated when participants were frustrated compared to a neutral affective state (Ihme et al., in press). However, the authors employed a manual technique for coding the facial muscle behavior and did not evaluate the potential of automatic frustration recognition. Based on these earlier results, we reasoned that *automated* recognition of frustration is possible by combining lip, mouth and eye brow movements.

Still, the goal of this work is not only to evaluate whether it is possible to discriminate frustrated from non-frustrated drivers based on video recordings from the face, but also to describe the patterns of facial muscle configuration related to frustration. For this, we used a multistep approach. First of all, we used a tool to extract the activity of facial action units (AUs) frame by frame from video recordings of the face (we used a commercial tool based on Bartlett et al., 2008). AUs are concepts from the Facial Action Coding System (FACS, Ekman et al., 2002) that can be regarded as the atomic units of facial behavior related to activation of facial muscles. The frame-wise activations of the facial AUs were then used as an input for time-resolved prediction of participants’ frustration using a machine learning approach which served to evaluate whether an automated discrimination of frustration is possible. In a second step, we aimed to identify the AU activations patterns that are indicative for frustration. For this, we clustered the frame-wise AU data in order to derive frequently occurring facial muscle configurations. Because facial expressions are described as momentary configurations of AU activations (Ekman, 1993), the resulting cluster centroids can be interpreted as representations of the frequently occurring facial expressions. The AU activations in the cluster centroids are then used to describe which AUs are activated and compared with previous results on facial expressions of frustration (D’Mello et al., 2005; Grafsgaard et al., 2013; Ihme et al., in press). In this way, we can determine which facial expressions are shown by frustrated drivers and whether these are in line with our expectation that facial expressions of frustration are related to lip, mouth and eye brow movements.

Only few studies investigated neural correlates of frustration despite it being a common emotional state. A functional magnetic resonance imaging (fMRI) study by Siegrist et al. (2005) investigated chronic social reward frustration experienced in a mental calculation task and revealed neural correlates of reward frustration primarily in the medial prefrontal, anterior cingulate and the dorsolateral prefrontal cortex (DLPFC). Bierzynska et al. (2016) induced frustration in a somatosensory discrimination task. The fMRI results revealed increased activation in the striatum, cingulate cortex, insula, middle frontal gyrus and precuneus with increasing frustration. Another fMRI study from Yu et al. (2014) using speeded reaction times found that experienced frustration correlated with brain activation in PFC and in deep brain structures like the amygdala, and the midbrain periaqueductal gray (PAG). These authors suggested that experienced frustration can serve as an energizing function translating unfulfilled motivation into aggressive-like surges via a cortical, amygdala and PAG network (Yu et al., 2014). Interestingly, other fMRI studies suggested a role of the anterior insula in the subjective experience of feeling frustrated (Abler et al., 2005; Brass and Haggard, 2007). Together, these fMRI studies provided detailed anatomical information about potential neural correlates of frustration, but mostly employed relatively simple experimental paradigms to induce frustration. Thus, in our study, we considered it desirable to employ a brain imaging technology that is better compatible with real-world applications in vehicles.

Functional near-infrared spectroscopy (fNIRS) is a non-invasive optical imaging technique that uses near-infrared light (600–900 nm) to measure cerebral blood flow changes when neural activity is elicited (Jöbsis, 1977; Villringer et al., 1993) based on neurovascular coupling similar to fMRI. fNIRS can be used to measure brain activation in realistic driving simulations and is relatively robust to movement artifacts (Unni et al., 2017). However, fNIRS measurements focus on cortical activation and their spatial resolution (around 3 cm) is lower than fMRI. Perlman et al. (2014) recorded fNIRS data from prefrontal cortical areas in 3–5-year-old children while they played a computer game where the expected prize was sometimes stolen by an animated dog to induce frustration. The results suggest a role for the lateral PFC in emotion regulation during frustration. Hirshfield et al. (2014) induced frustration by slowing down the internet speed while participants performed the task of shopping online for the least expensive model of a specified product given limited time constraints. The fNIRS results indicate increased activation in the DLPFC and the middle temporal gyrus when frustrated. A more recent study by Grabell et al. (2018) investigated the association between the prefrontal activation from fNIRS measurements and irritability scores in children. These authors reported an inverted U-shaped function between the children’s self-ratings of emotion during frustration and lateral prefrontal activation such that children who reported mild distress showed greater activation than peers who reported no or high distress highlighting the role of the lateral prefrontal areas and their involvement in emotion regulation. In sum, fNIRS and fMRI neuroimaging studies revealed that activation in prefrontal cortices plus several other brain areas, potentially specific to the exact task demands, are modulated by frustration. Based on this, in our study, we hypothesize that the lateral prefrontal areas might be indicative of frustration while driving.

To the best of our knowledge, no study exists that investigated the brain activation of drivers experiencing frustration and that uses brain activity measured with fNIRS for automated recognition of frustration. In addition, we are not aware of any study that aimed at continuous, time resolved prediction of driver frustration from facial expression or brain activation measurements. Therefore, the goals of this study were to evaluate whether it is possible to detect spontaneously experienced frustration of drivers based on: (1) video recordings of the face; and (2) brain activation as measured with fNIRS. In addition, we aimed to reveal facial muscle features and cortical brain activation patterns linked to frustration. To this end, we conducted two driving simulator experiments, in which frustration was induced through a combination of time pressure and blocking a goal, while videotaping the faces of the participants (Experiment 1) and recording brain activation using fNIRS (Experiment 2). We employed a multivariate data-driven approach to evaluate whether a discrimination of frustration from a non-frustrated state is possible using the data at hand. This analysis provided us with an estimate for the discriminability of the two induced affective states but could not tell about the underlying patterns of facial and brain activity that are related to frustration. Therefore, additionally, we investigated the underlying facial expressions and brain activity patterns in a second step and report the results thereof.

## Materials and Methods

### Experiment 1

#### Participants

Thirty-one volunteers participated in Experiment 1. The video recording of one participant failed due to a technical problem. Consequently, the data of 30 participants (twelve females, age mean [M] = 26.2 years, standard deviation [SD] = 3.5 years) were included in the analysis. All participants held a valid driver’s license, gave written informed consent prior to the experiment and received a financial compensation of 21 € for their participation. The experiments of this study were carried out in accordance with the recommendations of the guidelines of the German Aerospace Center and approved by the ethics committee of the Carl von Ossietzky University, Oldenburg, Germany. All subjects gave written informed consent in accordance with the Declaration of Helsinki.

#### Experimental Set-Up

The study was accomplished in a driving simulator consisting of a 46-inch screen (NEC MultiSync LCD4610) with a resolution of 1366 × 768 pixels, a G27 Racing gaming steering wheel (Logitech, Newark, CA, USA) including throttle and brake pedal and a gaming racing seat. Via the steering wheel and the pedals, the participants could control a virtual car in a driving simulation (Virtual Test Drive, Vires Simulationstechnologie, Bad Aibling, Germany). Sounds of the driving simulation were presented via loudspeakers (Logitech Surround Sound Speakers Z506). During the experiment, the participant’s face was filmed using a standard IP-Camera (ABUS, Wetter, Germany) with a resolution of 1280 × 720 pixels at a sampling rate of 10 frames per second.

#### Experimental Design and Cover Story

Frustration is experienced when goal-directed behavior is blocked (Lazarus, 1991) and can be intensified by time pressure (e.g., Rendon-Velez et al., 2016). Therefore, a cover story was created that told the participants to imagine being a driver at a parcel delivery service and having to deliver a parcel to a client (goal-directed behavior) within 6 min (time pressure). Participants were told that they received 15 € reimbursement for the experiment plus a bonus of 2 € for every parcel delivered within the given time. The drives took place in a simulated urban environment with two lanes (one per direction). Participants were told to stick to the traffic rules and to not exceed the speed limit of 50 km/h (~31 mph, which is the standard speed limit in urban areas in Germany). The experiment started with a short training with moderate traffic which lasted about 10 min. All participants drove the drives of all conditions (within-subjects block design), which are specified in the following sections.

##### Frust Condition

Three drives were used to induce frustration. In these, the participants had to deliver the parcel in 6 min, but their driving flow was blocked by events on the street. It was tried to distribute the occurrence of the frustrating events roughly equal over time in the scenario. However, the exact timing differed and also depended on participants’ speed. In addition, the nature of the events was varied (e.g., red lights, construction sites, slow lead cars that could not be overtaken, or a pedestrian crossing the street slowly) with the goal to create a scenario feeling as natural as possible to the participant. In two Frust drives, the participants were told after 6 min that the parcel could not be delivered in time, they will win no extra money and they should stop the car (of these two drives, the first drive had six frustrating events and the second one had eight). In the third drive participants were told after 5:40 min that they successfully delivered the parcel and won 2 € extra (this drive had eight frustrating events).

##### NoFrust Condition

Three further drives served as control condition. The participants had to deliver the parcel in 6 min with only little or moderate traffic on the ego lane (i.e., the lane they drove on), so that driving at the maximally allowed speed was almost always possible. In two of the drives, the participants were told after a fixed amount of time below 6 min (5:41 and 5:36 min) that they successfully delivered the parcel and won 2 € extra. The third non-frustrated drive ended after 6 min with a message that the time is over and no extra money was won.

The design of the frustrating drives was similar to the experimental manipulations of earlier studies on driver frustration (Lee, 2010; Lee and LaVoie, 2014). The participants drove the experimental conditions in random order and were not informed whether the current drive was a Frust or a NoFrust drive, i.e., they only experienced more or less frequently blocking events during a given drive. In order to reduce carry-over effects between the experimental drives, the participants had to drive for about 2 min through the same urban setting without any concurrent traffic between two experimental drives. Between the drives, there were breaks, in which participants had to fill in the questionnaires mentioned below and could take some time to relax.

#### Subjective Rating

As a manipulation check, the participants rated their subjectively experienced emotion using the SAM (Bradley and Lang, 1994) after each drive. In addition, they filled in the NASA-Task Load Index (NASA-TLX) after each Frust and NoFrust drive (Hart and Staveland, 1988). Here, we specifically focused on the frustration scale. One participant did not fill in the NASA-TLX, so only 29 questionnaires could be analyzed for that scale.

#### Data Analyses

##### Subjective Rating

The subjective ratings for the three used questionnaire items were compared to each by means of analysis of variance (ANOVA). Partial eta-squared ($\eta_{\text{p}}^{2}$) was calculated for each test as an indicator for effect size.

##### Pre-processing of Video Data

The software FACET (Imotions, Copenhagen, Denmark), which is based on the CERT toolbox (Bartlett et al., 2008), was used to extract information regarding the frame-wise AU activity. FACET makes use of the FACS (Ekman et al., 2002) and can determine the activation of 18 AUs as well as head motion (pitch, roll and yaw in °). An overview of the AUs recorded by FACET can be found in Table 1. The activation of AUs is coded as evidence, which indicates the likelihood of activation of the respective AU. For instance, an evidence value of 0 means that the software is uncertain about whether or not the respective AU is activated, a positive evidence value refers to an increase in certainty and a negative value to decreasing certainty. In order to reduce inter-individual difference in the evidence value, we subtracted the mean evidence value of the first minute of each drive per AU from the remaining values. In addition, a motion correction was accomplished as FACET operates optimally if the participants’ face is located frontally to the camera. Therefore, we analyzed only the frames with a pitch value between −10° to 20° as well as roll and yaw values between −10° and +10°. About 10.6% of the data were removed in this step.

**Table 1:** Overview of recorded action units (AUs).

AU	Description
1	Inner brow raiser
2	Outer brow raiser
4	Brow lowerer
5	Upper lid raiser
6	Cheek raiser
7	Lid tightener
9	Nose wrinkler
10	Upper lip raiser
12	Lip corner puller
14	Dimpler
15	Lip corner depressor
17	Chin raiser
18	Lip puckerer
20	Lip stretcher
23	Lip tightener
24	Lip pressor
25	Lips part
28	Lip suck

*The list and the descriptions of the AUs have been taken from the website of Imotions (Farnsworth, 2016)*.

##### Multivariate Cross-Validated Prediction of Frust and NoFrust Drives Based on AU Data

We used a multivariate logistic ridge regression (Hastie et al., 2009) decoding model implemented in the Glmnet toolbox (Qian et al., 2013) for the prediction of Frust and NoFrust drives from the *z*-scored AU activation (i.e., time resolved evidence). A 10-fold cross-validation approach was used to validate the model. For this, the time series data were split into 10 intervals. This approach avoids overfitting of the data to the model and provides an estimate of how well a decoding approach would predict new data in an online analysis (Reichert et al., 2014). In the logistic ridge regression, the λ parameter (also as hyper-parameter) determines the overall intensity of regularization. We used a standard nested cross-validation procedure to train the model and test generalization performance. The λ parameter was optimized in an inner 10-fold cross-validation loop implemented in the training. The outer cross-validation loop tested the generalization of the regression model with the optimized λ on the held-out test dataset. The input features that went into the decoding model were the pre-processed AU activations averaged across 10 data frames (= 1 s, no overlapping windows) to reduce the amount of data and increase the signal to noise ratio without increasing the model complexity. The model weights these input features and provides an output which is between 0 and 1. This output value indicates the likelihood for the test data classified as either the Frust class or the NoFrust class. An output ≥0.5 is considered to be classified as Frust drive whereas an output <0.5 is considered to be classified as a NoFrust drive.

The accuracy of the classification model of the individual participants was calculated as follows:

(1) $$\begin{array}{l} \text{Model Accuracy (\%) =}\\ \frac{TPR_{\text{Frust}}+TPR_{\text{NoFrust}}}{TPR_{\text{Frust}}+TPR_{\text{NoFrust}}+FPR_{\text{Frust}}+FPR_{\text{NoFrust}}}*100 \end{array}$$

In Equation 1, the TPR is the true positive rate and FPR is the false positive rate of the two conditions as denoted by Frust or noFrust. The model accuracy by itself is not a sufficient measure for evaluating the robustness of the model. Other performance measures like recall and precision are important indicators to evaluate whether the model exploits group information contained in the data and are insensitive to group size differences (Rieger et al., 2008). Recall is the proportion of trials which belong to a particular empirical class (Frust or NoFrust) and were assigned to the same class by the model. Precision provides information about how precise the model is in assigning the respective class (Frust or NoFrust). In this study, we report the F1-score, which is the harmonic average of precision and recall. An F1-score of 1 indicates perfect precision and recall (Shalev-Shwartz and Ben-David, 2016). The F1-score for the Frust condition was calculated as follows:

(2) $$\text{F}1\text{-score=}\frac{2*TPR_{\text{Frust}}}{2*TPR_{\text{Frust}}+FPR_{\text{Frust}}\_FPR_{\text{NoFrust}}}$$

##### Characterization of Facial Activation Patterns of Frustration: Clustering

A clustering approach was employed to identify patterns of co-activated AUs frequently occurring in the Frust and in the noFrust drives and to compare these between the two conditions. These patterns of co-activated AUs can be seen as the most frequently shown facial expressions during the driving scenarios. For the clustering, we separated the AU data in two sets: a training data set that contained two randomly selected drives from the Frust condition per participant and a test set including the remaining Frust drives and one randomly selected noFrust drive. For the cluster analysis, we used the data of all participants to ensure sufficient sample size. A recent work that simulated the effect of sample size on the quality of the cluster solution recommends using at least 70 times the number of variables considered (Dolcinar et al., 2014). The number of sample points in our training data set was roughly 14 times higher than this recommendation (30 participants × 2 drives × 5 min × 60 s = 18,000 data points >70 × 18 AUs = 1,260). K-means clustering with *k* = 5 was conducted on the training set. A value of *k* = 5 was chosen after visually inspecting a random selection of video frames of the face recordings. It seemed as if five different expressions were shown predominantly. We applied the resulting cluster centroids to cluster the data from the test set (i.e., each data point was assigned to the cluster with the smallest distance to the centroid). From this, we could determine the percentage of data points per condition assigned to each of the five clusters (per participant) and compare the conditions by means of paired Wilcoxon tests. In addition, we characterized the resulting clusters by their patterns of activated AUs in the centroids. An AU was assumed to be activated if the evidence in the centroid was ≥0.25. This criterion was adopted from Grafsgaard et al. (2013), who used the same threshold to select activated AUs in their work. We report the five resulting clusters with the AUs that characterize these as well as the results of the Wilcoxon test. Moreover, we investigated the relationship between the subjectively reported frustration levels (by means of the NASA-TLX frustration item) and the probability of the clusters in the test set. For this, we correlated both values with each other using Kendall’s Tau (as the data were not normally distributed). In order to account for the variability between subjects, we additionally performed a linear mixed effects analysis of the relationship between the probability of Cluster 4 and the subjective frustration rating using the combined data of training and test set. With this, we wanted to estimate whether we can predict the probability of showing Cluster 4 using the subjective frustration rating as fixed effect. As random effects we had intercepts for participants and by-participant random slopes for the effect of the subjective rating. *P*-values were obtained by likelihood ratio tests (*χ*^2^) of the full models with the effect in question against the models without the effect in question (see Winter, 2013). The models were calculated using the R package lme4 (Bates et al., 2015).

### Experiment 2

#### Participants

Sixteen male volunteers aged between 19 and 32 (*M* = 25.3, *SD* = 3.5) years participated in Experiment 2. All participants possessed a valid German driving license and provided written informed consent to participate prior to the experiment. They received a financial compensation of 30 € for the participation in the experiment. The data from one participant was excluded because the participant suffered from simulation sickness during the course of the experiment. Data from three other participants were excluded due to a large number (>50%) of noisy channels in the fNIRS recordings. The mean age of the remaining participants was 25.2 (*SD* = 3.8) years. This study was carried out in accordance with the recommendations of the guidelines of the German Aerospace Center and approved by the ethics committee of the Carl von Ossietzky University, Oldenburg, Germany. All subjects gave written informed consent in accordance with the Declaration of Helsinki.

#### Experimental Set-Up

The experiment was accomplished in the virtual reality (VR) lab with 360° full view at the German Aerospace Center (Fischer et al., 2014). Participants sat in a realistic vehicle mock-up and controlled the mock-up car in the driving simulation (Virtual Test Drive, Vires Simulationstechnologie, Bad Aibling, Germany) via a standard interface with throttle, brake pedal, steering wheel and indicators.

#### Experimental Design and Cover Story

The same parcel delivery service cover story as in Experiment 1 was used. The only difference was that the participants received a slightly higher basic reimbursement of 18 € (instead of 15 € in Experiment 1) due to the longer overall duration of the experiment. The bonus of 2 € for every parcel delivered within the given time was the same as in Experiment 1. In the end, all participants were paid 30 € for their participation, irrespective of their success. The experiment was structured as a block design and began with a short training of roughly 10 min with moderate traffic. Thereafter, we recorded the baseline data for 2 min following which the participants drove the Frust and noFrust drives (six per condition) in alternation on the same urban track as in Experiment 1. The order of each type of drives was randomized. The experimental conditions are specified in the following sections.

##### Frust Condition

In the Frust drives, the participants had to deliver the parcels within a maximum time of 6 min, but their driving was blocked by events on the street (similar to Experiment 1, but with a bit less complexity, e.g., no pedestrians involved). The blocking events had an average time distance of 20 s (i.e., after 20 s of driving, an obstacle occurred). There were seven blocking events per drive. However, if the participant drove very slowly, it could be that less blocking events were passed. In case they reached the goal within 6 min, a message was presented telling them that they received 2 €. If they did not reach the goal after 6 min, they were informed that they did not succeed this time. Both messages ended the drives accordingly.

##### NoFrust Condition

The noFrust drives served as control condition. Participants were told that they had to pick up the parcels from headquarters. There was moderate traffic on the ego lane, so that driving at the maximally allowed speed was almost always possible. The drives took 5 min. Between the drives, there were breaks, in which participants had to fill in the questionnaires mentioned below and could take some time to relax.

#### Subjective Rating

As a manipulation check, the participants rated their subjectively experienced emotion using the SAM (Bradley and Lang, 1994) after each drive.

#### fNIRS Set-Up

**Figure 1:**
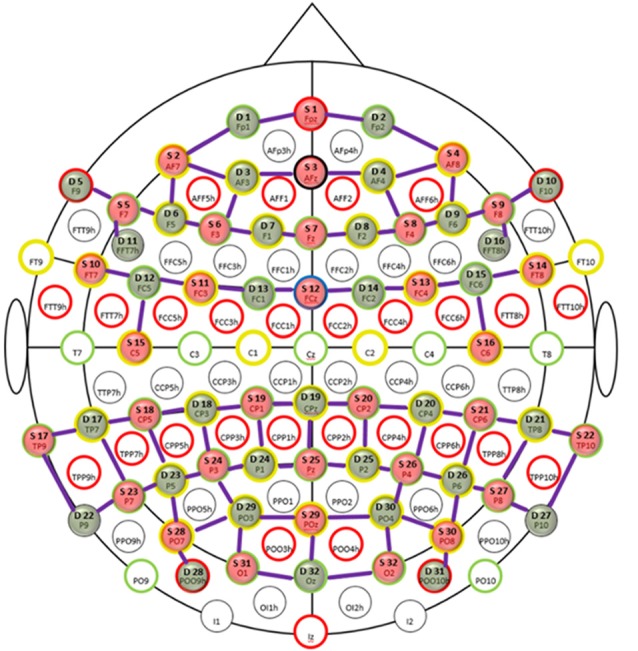
Probe placement of functional near-infrared spectroscopy (fNIRS) channels. Topologic layout of emitters (red), detectors (green) and fNIRS channels (purple) with coverage over the frontal, parietal and temporo-occipital cortices superimposed on a standard 10-20 EEG layout. The anatomical markers are highlighted by red, green, yellow and black circles. The blue circle marks the frontal central midline (FCz) sagittal plane. Figure reproduced from NIRStar software with permission from NIRx Medical Technologies, LLC, Minneapolis, MN, USA.

Functional near infrared spectroscopy is a non-invasive optical imaging technique that uses near-infrared light (600–900 nm) to measure hemodynamic responses in the brain (Jöbsis, 1977; Villringer et al., 1993). This is done by measuring the absorption changes in the near-infrared light that reflects the local concentration changes of oxy-hemoglobin (HbO) and deoxy-hemoglobin (HbR) in the sub-cortical brain areas as correlates of functional brain activity. We recorded fNIRS data from the frontal, parietal and temporo-occipital cortices using two NIRScout systems (NIRx Medical Technologies GmbH, Berlin, Germany) in tandem mode resulting in 32 detectors and emitters at two wavelengths (850 and 760 nm). In total, we had 80 channels (combinations of sources and detectors) each for HbO and HbR as shown in Figure 1. The distances for the channels ranged between 2 cm and 4 cm (*M* = 3.25, *SD* = 0.45). The shortest channels were the source-detector combinations S5-D11 and S9-D16 in the bilateral prefrontal areas whereas the longest channels were S25-D19 in the parietal midline and S28-D29 and S30-D30 in the bilateral occipital areas (see Figure 1). To ensure that the fNIRS cap was placed in a reliable way across all participants, we checked if the position of the optode holder on the fNIRS cap for the anatomical location Cz on the midline sagittal plane of the skull is equidistant to the nasion and the inion and equidistant to the ears. The sampling frequency of the NIRS tandem system was almost 2 Hz.

#### Data Analyses

##### Subjective Rating

The subjective ratings for the two questionnaire items were compared to each by means of ANOVA. Partial eta-squared effect sizes ($\eta_{\text{p}}^{2}$) were calculated for each test.

##### fNIRS Data Pre-processing

The raw data from fNIRS measurements record the influence of not only cortical brain activity but also other systemic physiological artifacts (cardiac artifacts, respiration rate, Mayer waves) and movement artifacts causing the signal to be noisy. To reduce the influence of these artifacts, the raw data was pre-processed using the nirsLAB analysis package (Xu et al., 2014). We first computed the coefficient of variation (CV) which is a measure for the signal-to-noise ratio (SNR) from the unfiltered raw data using the mean and the standard deviation of each NIRS channel over the entire duration of the experiment (Schmitz et al., 2005; Schneider et al., 2011). All channels with a CV greater than 20% were excluded from further analysis. Additionally, we performed a visual inspection and deleted channels which were excessively noisy with various spikes. On average, 64 channels each for HbO and HbR per participant and were included in the analysis (*SD* = 7.46). We then applied the modified Beer-Lambert’s law to convert the data from voltage (μV) to relative concentration change (mmol/l; Sassaroli and Fantini, 2004).

To reduce effects of movement artifacts and systemic physiology, we used an autoregressive model of order *n* (AR(*n*); *n*_max_ = 30) based on autoregressive iteratively reweighted least squares developed by Barker et al. (2013) implemented as a function in the nirsLAB 2017.6 Toolbox^1^. The algorithm fit the residuals of each individual channel to an AR(*n*) model, where *n* is the order that minimized the Bayesian information criterion. With the resulting autoregressive coefficients, a pre-whitening filter was generated that was applied to the fNIRS data. The reason for that is the fact that fNIRS time series data are typically characterized by large outliers caused by movement artifacts and serially correlated noise from the physiological artifacts and the temporal correlation of the time samples. This generally leads to incorrect estimation of regressor weights when performing univariate regression analyses and results in overestimation while computing corresponding statistical values, causing an increase in false positives and false negatives (Tachtsidis and Scholkmann, 2016). Pre-whitening can handle such noise correlated time series data where an autoregressive model takes into account the correlation between the current time sample and its neighboring samples and models the temporal correlations.

##### Multivariate Cross-Validated Prediction of Frust and NoFrust Drives From fNIRS Data

In line with Experiment 1, we used the multivariate logistic ridge regression (Hastie et al., 2009) decoding model implemented in the Glmnet toolbox (Qian et al., 2013) for the prediction of Frust and NoFrust drives from sample-by-sample fNIRS brain activation data. The input features that went into the decoding model were the pre-processed HbO and HbR values which were *z*-scored for the particular segments of Frust and noFrust drives. Both HbO and HbR features were used simultaneously. The model weighted these input features and provided an output between 0 and 1. This output value indicates the likelihood for the test data classified as either the Frust class or the NoFrust class. Like in Experiment 1, accuracy and F1 score are reported to estimate the model prediction.

##### Characterization of Brain Areas Predictive to Frustration: Univariate Regression Analysis

In order to characterize the pattern of brain areas involved during frustrating drives, we performed univariate regression analyses on a single-subject level separately for each fNIRS channel using the generalized linear model (GLM) analysis module implemented in the nirsLAB Toolbox. Our design matrix consisted of two regressors that corresponded to the entire blocks of Frust and noFrust drives. The autoregressive model AR(*n*) from Barker et al. (2013) that generated the pre-whitening filter and was applied to the fNIRS time-series data was also applied to the design matrix. Regression co-efficients were estimated by convolving a boxcar function weighted corresponding to the entire blocks of Frust and noFrust drives with a canonical hemodynamic response function (HRF) implemented in the nirsLAB toolbox (Xu et al., 2014) which composed of one gamma function for HbO. The time parameters at which the response reached the peak and undershoot were 6 s and 16 s, respectively. The canonical HRF was reversed for HbR in order to match the effect-sizes for HbO and HbR brain maps for a particular contrast in the same direction since the HbO and HbR signals are correlated negatively. This setting is applied by default in nirsLAB while estimating the GLM co-efficients for HbR. Channel-wise beta values were used to compute t-statistic for each channel separately for the contrast (difference: Frust-noFrust). Finally, we performed a group level analysis for generalization of the brain areas predictive to frustration while driving. The beta values computed from GLM for each channel and each participant in the individual analysis was used for the group-level analyses. The group-level analyses represented the standard deviation for the beta values for each channel across participants.

## Results

### Experiment 1

#### Subjective Rating

The participants rated the drives from the Frust condition as significantly more arousing, more negative and more frustrating than the drives from the NoFrust condition. The results of the subjective rating are presented in Table 2. The frustration rating showed a strong negative correlation with the valence rating (*r* = -0.61, *p* < 0.001) and a marginal significant positive correlation with the arousal rating (*r* = 0.24, *p* = 0.06). Valence and arousal were negatively correlated (*r* = −0.46, *p* < 0.001).

**Table 2:** Means (M), standard deviations (SD) and results of the analysis of variance (ANOVA) for the subjective ratings (self-assessment manikin [SAM] valence, SAM arousal and NASA Task Load Index [NASA-TLX] frustration score).

	Frust		noFrust		ANOVA			
	*M*	*SD*	*M*	*SD*	*F*	*df*	*p*	$\eta_{\text{p}}^{2}$
SAM arousal	4.9	1.6	3.9	1.6	14.67	(1, 29)	<0.01*	0.34
SAM valence	0.0	1.3	1.8	1.2	73.06	(1, 29)	<0.001*	0.72
NASA-TLX frustration	6.3	2.2	4.4	2.0	33.70	(1, 28)	<0.001*	0.55

Note that one participant failed to fill in the NASA-TLX which explains the reduced number of degrees of freedom (df) for that item. Higher values indicate higher arousal (1–9), higher valence (−4 to +4) and higher frustration (1–9). Significant results are marked with a “*”.

#### Multivariate Prediction of Frust and NoFrust Drives Based on AU Data

**Figure 2:**
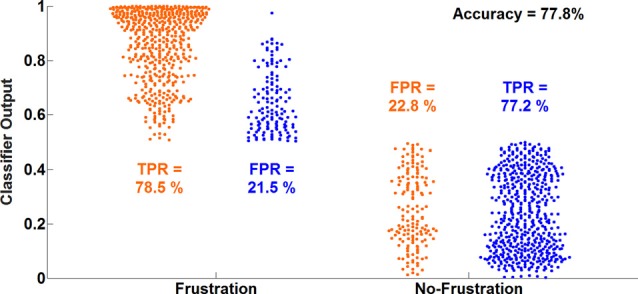
Ten-fold cross-validated prediction of frustrating and non-frustrating drives from action unit (AU) activations using multivariate logistic ridge regression analysis for an example participant (P11). The data of this participant allowed the classification with the highest accuracy.

The average classification accuracy for the Frust vs. the noFrust condition using the multivariate approach based on the AU activations was 62.0% (*SD* = 9.6%) and the mean F1 score was 0.617 (*SD* = 0.097). The individual classification results for each participant from the 10-fold cross-validation are presented in Table 3. Figure 2 depicts the results from the multivariate logistic ridge regression model from the participant with the highest classification accuracy of 77.8% (participant 11). The results presented in this figure show the model output for all test data. In Figure 2, each orange sample point is the output of the decoding model for the AU test data seen from the Frust drives. Similarly, each green sample point is the output of the model for the AU test data as seen from the noFrust drives. We present our results in the form of TPR (i.e., data from a particular drive is classified as the correct drive) and FPR (i.e., data from a particular drive is classified as the opposite drive). Here, one can see that a TPR of 78.5% and 77.2% and a FPR of 21.5% and 22.8% were achieved for the Frust and NoFrust drives respectively. For the example participant, an F1-score of 0.78 was achieved.

**Table 3:** Ten-fold cross-validated predictions of Frust and NoFrust drives from AU data using multivariate logistic ridge regression analysis for all participants.

Participant	P1	P2	P3	P4	P5	P6	P7	P8	P9	P10	P11	P12	P13	P14	P15
Mean accuracy (%)	63	59	58	55	49	54	78	78	56	60	78	70	70	56	58
F1-score	0.61	0.58	0.58	0.54	0.49	0.54	0.78	0.78	0.56	0.60	0.78	0.70	0.70	0.56	0.58
**Participant**	**P16**	**P17**	**P18**	**P19**	**P20**	**P21**	**P22**	**P23**	**P24**	**P25**	**P26**	**P27**	**P28**	**P29**	**P30**
Mean accuracy (%)	49	70	73	60	64	53	64	73	60	55	64	51	71	41	71
F1-score	0.49	0.70	0.72	0.60	0.65	0.53	0.63	0.73	0.59	0.53	0.64	0.51	0.72	0.40	0.70

#### Characterization of Facial Activation Patterns of Frustration: Clustering Approach

**Figure 3:**
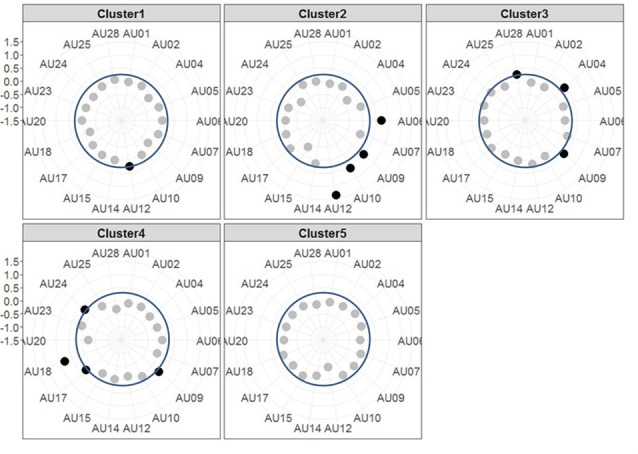
Radar plots showing the results of the k-means clustering. Each plot shows the centroids of one Cluster with each axis of the radar plot referring to the evidence of one AU. The dots mark the evidence for the respective AU, i.e., the further outside they are, the higher the evidence is (the axis for evidence ranges from −1.5 to +1.5 with each gray line indicating a step of 0.5). AUs that are considered as activated (i.e., with an evidence ≥0.25, indicated by blue circle line) are printed in black, the others in gray.

**Figure 4:**
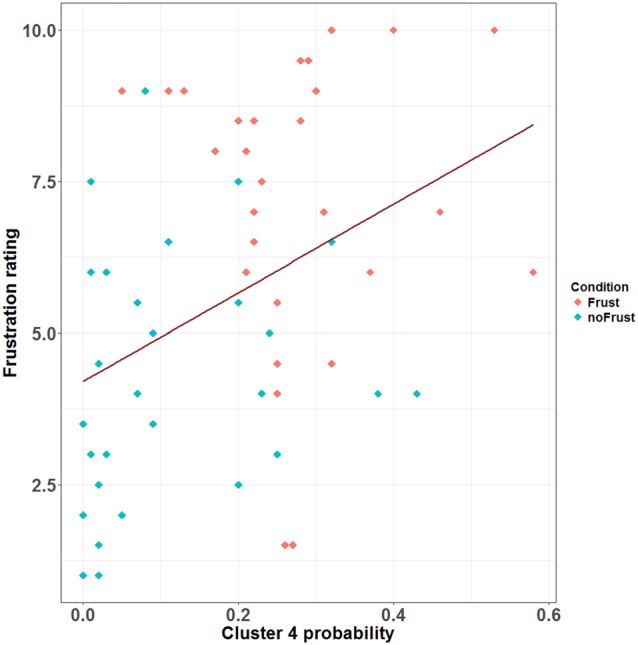
Scatterplot showing the correlation between the probability (relative frequency) of Cluster 4 and the subjective frustration rating. The plot contains data from the Frust (red) and noFrust (turquoise) condition. The regression line is plotted in dark red.

The resulting cluster centroids from the k-means clustering, which can be seen as the most frequently shown facial expressions during the drives, are presented in Figure 3. When applying the cluster centroids to the (unseen) test set, Cluster 4 and Cluster 5 displayed a significantly different relative frequency of occurrence (i.e., percentage of video frames attributed to this cluster centroid) between the two conditions. Specifically, Cluster 4 was found more often in the Frust condition (*M* = 27.3%, *SD* = 14.18%) compared to the noFrust condition (*M* = 10.9%, *SD* = 12.1%, *Z* = 3.95, *p* < 0.001) and Cluster 5 more often in the noFrust (*M* = 33.7%, *SD* = 19.9%) than the Frust condition (*M =* 22.7%, *SD* = 10.9%, *Z* = −1.87, *p* < 0.05). No significant differences were observed for the three other clusters (see Table 4). Cluster 4 is characterized by above threshold activity (i.e., evidence >0.25) in AU9 (nose wrinkler), AU17 (chin raiser), AU18 (lip pucker) and AU24 (lip pressor). In comparison, Cluster 5 accounts for no AU with above threshold evidence. The other clusters are described by different patterns of AU activity: Cluster 1 shows little activity in all AUs (only AU12 [lip corner puller] has evidence >0.25), Cluster 2 the highest activation in AU6 (cheek raiser), AU9 (nose wrinkler), AU10 (upper lip raiser) as well as AU12 (lip corner puller) and Cluster 3 mostly in AU4 (brow lowerer), AU9 (nose wrinkler) and AU28 (lip suck; see Table 5 for an overview and a possible interpretation). Interestingly, the correlation analysis revealed that the frequency of occurrence of the cluster that was shown more often in the Frust condition (Cluster 4) also positively correlated with the subjective frustration rating (τ = 0.27, *p* < 0.05, see Figure 4). No other cluster showed a significant relationship with the subjectively experienced frustration (Cluster 1: τ = 0.13, *p* = 0.15, $\chi_{\left(1\right)}^{2}$ = 3.57, *p* = 0.06; Cluster 2: τ = 0.02, *p* = 0.85, $\chi_{\left(1\right)}^{2}$ = 0.75, *p* = 0.38; Cluster 3: τ = −0.03, *p* = 0.75, $\chi_{\left(1\right)}^{2}$ = 0.03, *p* = 0.86; Cluster 5: τ = 0, *p* = 0.98, $\chi_{\left(1\right)}^{2}$ = 0.03, *p* = 0.87). The positive relationship between the frustration rating and probability of Cluster 4 was confirmed by results of the linear mixed effects analysis including intercepts for participants and by-participant random slopes as random effects, which revealed a significant relationship between the subjective frustration rating and Cluster 4 probability ($\chi_{\left(1\right)}^{2}$ = 6.74, *p* < 0.01). The analysis of the fixed effect rating revealed that each increase of the subjective frustration rating by 1 on the scale increased the probability of showing Cluster 4 by 1.5% (standard error: 0.5%). Comparing the model with a simpler model without inclusion of random effects revealed that the Akaike information criterion (AIC, Akaike, 1998) was lower for the model with random effects (AIC = −110.2) compared to the one without the random effects (−95.5). This suggests that the model better explains the data if the random effects are included.

**Table 4:** Relative frequency of attribution of data points from the test set to the cluster centroids extracted from the test set for the two conditions Frust and noFrust.

	Frust		noFrust		Wilcoxon test	
	*M*	*SD*	*M*	*SD*	*Z*	*p*
Cluster 1	20.6	13.4	22.6	16.9	−0.32	0.63
Cluster 2	10.3	16.9	10.1	11.0	1.40	0.08
Cluster 3	19.2	14.8	22.8	12.1	0.03	0.49
Cluster 4	27.3	14.8	10.9	12.1	3.95	<0.001*
Cluster 5	22.7	10.9	33.7	19.9	−1.87	<0.05*

*Means (M), standard deviations (SD) and results of Wilcoxon test are presented. Significant differences are marked with an asterisk*.

**Table 5:** Description of the five clusters including the involved AUs and a potential interpretation of the meaning.

Cluster	Involved action units	Potential interpretation
1	AU12	neutral to slight smile
2	AU6, AU9, AU10, AU12	smiling
3	AU4, AU9, AU28	frowning
4	AU9, AU17, AU18, AU24	frustrated
5	No above threshold AU	neutral

### Experiment 2

#### Subjective Ratings

In concordance with the results of Experiment 1, the participants rated the Frust drives as more arousing (SAM arousal, Frust: *M* = 4.7, *SD* = 1.4; noFrust: *M* = 3.5, *SD* = 1.1, *F*_(1,11)_ = 26.87, *p* < 0.01, $\eta_{\text{p}}^{2}$ = 0.71) and more negative (SAM valence, Frust: *M* = 0.75, *SD* = 1.2; noFrust: *M* = 1.5, *SD* = 1.0, *F*_(1,11)_ = 15.67, *p* < 0.01, $\eta_{\text{p}}^{2}$ = 0.59) than the noFrust drives. Valence and arousal were negatively correlated (*r* = 0.55, *p* < 0.01).

#### Multivariate Prediction of Frust and NoFrust Drives From fNIRS Data

The mean frustration prediction accuracy and F1-score obtained with fNIRS brain activation recordings across all participants were 78.1% (*SD* = 11.2%) and 0.776 (*SD* = 0.115), respectively. Table 6 lists the individual results for all participants.

**Table 6:** Ten-fold cross-validated predictions of Frust and NoFrust drives from fNIRS measurements using multivariate logistic ridge regression analysis for all participants.

Participant	P1	P2	P3	P4	P5	P6	P7	P8	P9	P10	P11	P12
Mean accuracy (%)	76	75	73	88	65	95	78	95	64	76	88	64
F1-score	0.76	0.74	0.73	0.88	0.64	0.95	0.77	0.95	0.63	0.76	0.87	0.63

**Figure 5:**
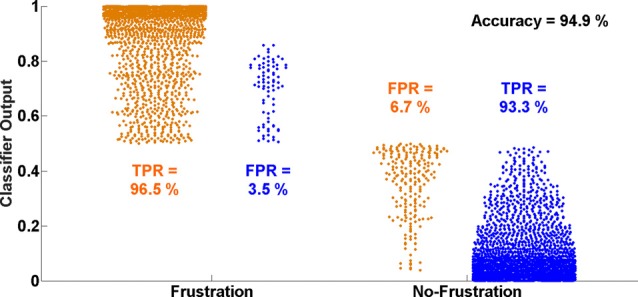
Ten-fold cross-validated prediction of Frust and NoFrust drives from deoxy-hemoglobin (HbR) and oxy-hemogflobin (HbO) fNIRS measurements using multivariate logistic ridge regression analysis for an example participant (P8).

Figure 5 shows the distributions of single time interval predictions of the multivariate logistic ridge regression model for the participant with the highest classification accuracy of almost 95% for HbR and HbO data. In this participant, a TPR of 96.5% and 93.3% and a FPR of 3.5% and 6.7% were achieved for the Frust and NoFrust drives, respectively.

#### Characterization of Brain Areas Predictive to Frustration

We performed univariate GLM analyses separately for each channel in order to determine the localization of brain areas most predictive to frustration while driving. The univariate approach was chosen because the model weights of the multivariate fNIRS regression model are hard to interpret for various reasons (Reichert et al., 2014; Weichwald et al., 2015; Holdgraf et al., 2017).

**Figure 6:**
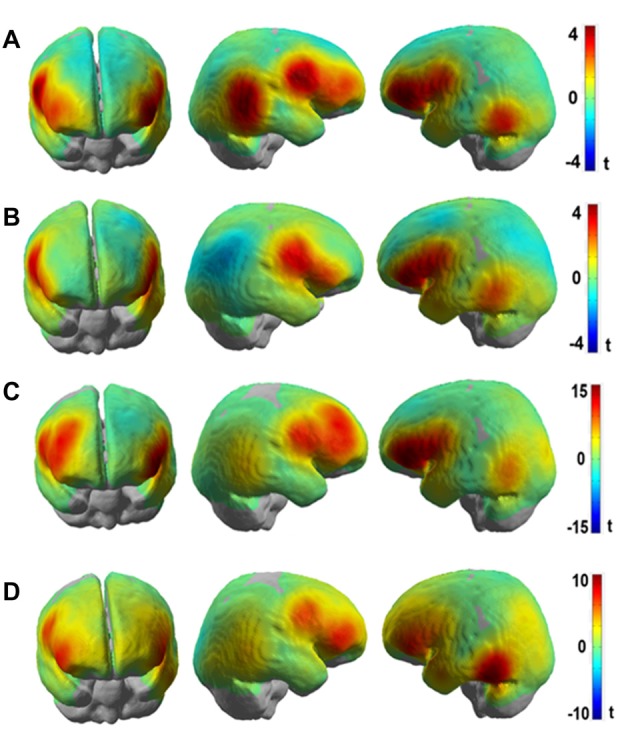
*t*-value maps obtained from channel-wise linear regression of **(A)** HbR and **(B)** HbO fNIRS data for the group-level analyses and channel-wise linear regression of **(C)** HbR and **(D)** HbO fNIRS data for single participant (P8, same participant as in Figure 5) for the (Frust- noFrust) condition using Generalized Linear Model (GLM). High positive *t-values* are indicated by red color, high negative values by blue color. Note that the activation of HbO and HbR maps are in the same direction in spite of the signals being negatively correlated, because the hemeodynamic response function was reversed for estimating the GLM co-efficients for HbR.

Figures 6A,B show the results presented as unthresholded *t*-value maps (difference: Frust-noFrust) from the channel-wise linear regression of HbR and HbO data for the group level analysis. The *t-value* maps indicate the local effect sizes, in essence they are Cohen’s d scaled by the square root of the number of samples included in their calculation. The *t-values* provide a univariate measure to estimate the importance of a feature for multivariate classification. The Bonferroni-corrected t-maps for the group-level analysis are included in the Supplementary Figures S1A,B. In Figures 6A,B, both HbR and HbO t-value maps show significant convergence in brain activation patterns bilaterally in the inferior frontal areas (putative BA45) and the ventral motor cortex (putative BA6). Additional informative channels can be seen in the right inferior parietal areas (putative BA22) only for the HbR maps but not for the HbO maps. This could be due to the averaging effects of the brain activation on a channel-level across participants who showed inter-individual variabilities. In both HbR and HbO maps, some channels in the left temporo-occipital areas (putative BA21) were found to be predictive to frustrated driving although the trend is not as strong there as it is in the frontal areas. Figures 6C,D show t-value maps for the same contrast from the channel-wise linear regression of HbR and HbO data for the participant with the highest prediction accuracies. These single participant brain activation patterns closely resemble the pattern of the group level map. However, the *t-values* are much higher in the single participant than in the group averaged map. Both, HbR and HbO signals indicate enhanced activation bilaterally in the inferior frontal and ventral motor areas (*t* > 10) during Frust drives in the single participant t-maps whereas the group averaged *t-values* rarely exceed *t* = 4. The reduced *t-values* in the averaged maps are due to variability of the predictive brain activation patterns with respect to both, spatial distribution and local effects sizes. This can be seen, for example, in the HbO t-statistic value maps which show predictive activation in the left inferior parietal and the left temporo-occipital areas in the single participant maps but less so in the group level analysis.

We visualized the averaged brain map on the MNI 152 brain in Neurosynth^2^ and used MRIcron^3^ to determine MNI co-ordinates and the corresponding Brodmann areas for the brain areas with increased activation differences between Frust and NoFrust drives. Table 7 lists the brain areas, the MNI-coordinates of the difference maxima and *t*-values as indicators of the effect sizes.

**Table 7:** Brain areas showing increased activation in the Frust compared to the noFrust condition.

Brain areas	Putative Brodmann Area (BA)	*X*	*Y*	*Z*	*t*-value
**For HbR**					
Right inferior frontal	45	46	48	24	2.0
Left inferior frontal	45	−49	41	24	3.6*
Right ventral motor	6	62	7	36	3.5*
Left ventral motor	6	−58	9	37	3.6*
Right inferior parietal	22	63	−46	11	4.2*
Left temporo-occipital	21	−63	−51	1	3.4
**For HbO**					
Right inferior frontal	45	51	31	16	2.2
Left inferior frontal	45	−53	35	20	4.1*
Right ventral motor	6	61	10	29	3.0
Left ventral motor	6	−59	10	36	3.4
Left temporo-occipital	21	−66	−47	7	2.3

The MNI coordinates of activation and their *t-values* are shown. *Indicates statistically significant differences which survived the Bonferroni-corrected thresholding of *p* < 0.05.

## Discussion

The goals of this study were to investigate discriminative properties of facial muscle activity extracted from video recordings and brain activation patterns using fNIRS for the automated detection of driver frustration. Therefore, two driving simulator experiments were conducted in which frustration was induced through a combination of time pressure and goal blocking. In Experiment 1, we videotaped the faces of the participants during the drives and extracted the activity of the facial muscles using automated video processing. We could show that the facial expression data can be used to classify frustration from a neutral state with an average classification accuracy of almost 62%. Frustration could be discriminated from a neutral state with above chance accuracy in most participants, with maximum accuracy up to 78% for the best participants. In addition, a detailed analysis comparing the muscle activation in both conditions revealed that the muscles nose wrinkler, chin raiser, lip pucker and lip pressor are activated in synchrony more often in the frustrating condition than in the neutral condition. The approach was then extended to fNIRS brain activation measurements in Experiment 2, where the discrimination of frustration from the neutral state improved to average classification accuracy of almost 78% and up to 95% for the best two participants. An additional univariate GLM analysis indicated that frustration during driving was reflected in reliable brain activation modulation bilaterally in ventrolateral inferior frontal areas in the group-level analysis. Our results demonstrate that frustration during driving could be detected time resolved from video recordings of the face and fNIRS recordings of the brain.

In both experiments, frustration was induced using a combination of events blocking goal-directed behavior and time pressure during simulated drives. According to research on frustration as well as previous studies on frustrated driving, this combination generally leads to a state of experienced frustration for the participants (Lazarus, 1991; Lee, 2010; Rendon-Velez et al., 2016). The accomplished manipulation checks showed that the participants rated the frustrating drives as more negative and more arousing than the non-frustrating drives in both experiments, which is in line with the classification of frustration in the valence-arousal space of emotions (Russell, 1980). Additionally, the participants assigned a higher score in the NASA-TLX frustration scale to the frustrating drives in Experiment 1. Therefore, we could conclude that the experimental manipulation indeed induced frustration and was suitable to study the proposed research questions.

Our approach of using multivariate logistic ridge regression in combination with cross-validation enabled us to explore the feasibility of using facial muscle activity extracted from video recordings of the face and almost whole-head fNIRS as an estimate for cortical activity for time-resolved characterization of driver frustration. The multivariate modeling allowed us to predict frustrated drives from the non-frustrated drives in a continuous manner with relatively high accuracy. For the facial muscle activation, the decoding model made predictions from the evidence values of 18 AU input features which were pre-selected with the used software. For the fNIRS brain activation, the input features to the decoding model were the sample-by-sample pre-processed fNIRS data from all the selected channels for each participant. On average, we had about 128 input features (*SD* = 14.9) across all participants. Our decoding models were able to discriminate driver frustration from non-frustration with a mean accuracy of 62% for facial muscle activation and almost 80% for cortical activation. The cross-validation approach allowed us to estimate the generalization of our decoding model to new data which our model had never seen before (i.e., the test dataset) indicating the true predictive power of our model necessary for online tracking of user states (Reichert et al., 2014; Holdgraf et al., 2017). The classification accuracy derived from the facial expression data is higher than chance level, but likely not high enough for robust usage in human-machine systems with adaptive automation. One reason for that may be the fact that humans do not show the same facial expression constantly over a period of several minutes, even though they report to be frustrated in that drive. Moreover, it is conceivable that the level of frustration also varied during the drives leading to fact that facial expression indicating other emotions or a neutral state may have been shown by participants. Together, this may have biased the training and test set as these not only included facial expressions of frustration, but also other facial expressions. This in turn could lead to the lower classification accuracy for facial expression data in comparison to the brain activation. Still, we can confirm our initial hypothesis that it is possible to discriminate driver frustration from a neutral affective state using facial muscle activity and cortical activation with above chance accuracy with cortical activation providing better classification results. It remains to be shown that the classification accuracy is high enough to ensure user acceptance in adaptive automation.

Since the supervised classification gives us only an estimate of how well we would be able to recognize frustration using the respective data frames, we also conducted a detailed analysis of the facial muscle and brain activation data to understand which features are indicative for frustration. For this, a clustering of the facial muscle activity was conducted in order to identify patterns of co-activated facial muscles that occur with increased likelihood if a driver is frustrated. The clustering approach revealed five different clusters of AU activity, which can be seen as the facial expressions that were shown (most frequently) during the drives. Cluster 4 was shown significantly more often in the Frust than in the noFrust condition and its probability additionally correlated with the subjective frustration rating. Therefore, this pattern subsuming activity from muscles from the mouth region (chin raiser, lip pucker and lip pressor) and traces from the nose wrinkle can likely be seen as comprising the frustrated facial expression. Interestingly, similar patterns of AU activation have been associated to frustration in previous research (D’Mello et al., 2005; Grafsgaard et al., 2013; Ihme et al., in press). In contrast, the Cluster 5 was activated more often in the non-frustrating drives by the participants. Because it also has no activated AUs involved, we consider it as referring to a neutral facial expression. None of the remaining clusters differed in frequency of occurrence between the two conditions. Presumably, Cluster 1 can also be seen as neutral, because it included only little facial muscle activity. With highest activation in cheek raiser and lip corner puller (and some activation in the nose wrinkler and the upper lip raiser), Cluster 2 likely represents a smiling face (Ekman and Friesen, 2003; Ekman, 2004; Hoque et al., 2012). Finally, Cluster 3 showed a pattern with high activity in action units around the eyes (brow lowerer and nose wrinkler), which could be a frowning as a sign of anger or concentration (Ekman and Friesen, 2003). One interesting issue is that the nose wrinkler (AU 9) occurred frequently and, according to our analysis, is part of Cluster 2 (smiling), 3 (frowning) and 4 (frustration), although most previous research has associated it predominantly with disgust (e.g., Ekman et al., 1980; Lucey et al., 2010), which was likely not induced through our experimental paradigm and set-up. We speculate that two aspects may explain this frequent occurrence of AU9. First, it could be that the software which we used misclassified movements of the eyebrows and attributed these to the nose wrinkler. This is possible and poses a disadvantage of automated techniques to extract facial muscle activity compared to manual coding approaches. Second, it could be that the nose wrinkler is not a particular sign of disgust, but rather a sign of one factor of a dimensional model of emotions. For example, Boukricha et al. (2009) have shown a correlation between AU9 and low pleasure as well as high dominance. We would like to stress here that although the frustrated facial expression (Cluster 4) occurred most often in the frustrated drives, the results indicate that it was not the only facial expression that has been shown by the participants (as already speculated above). Therefore, the approach to cluster time-resolved AU activations into patterns of co-activation in order to gain information about the shown facial expressions appears promising to better understand which facial expressions are shown by the drivers when they experience frustration or other emotions. Future studies should evaluate whether the results from the clustering can be utilized to generate labels that not only indicate the emotion induction phase from which a sample stems, but the facial expression that was actually shown by the participant. This could improve the training data set for the classification as well as classification accuracy. To sum up, the detailed cluster analysis revealed that the facial expression of frustration is mainly linked to the facial muscle activity in the mouth region.

To investigate the frustration predictive features from the fNIRS brain recordings, we performed univariate regression analyses separately for each channel using GLM to determine the localization of brain areas most predictive to frustration while driving. Our group level results indicate that fNIRS brain activation patterns of frustrated drivers were clearly discernible from non-frustrated drivers. Frustration during driving was reflected with stronger HbR and HbO activation bilaterally in the inferior frontal areas (putative BA 45) and the ventral motor cortex (putative BA 6) in the group level analysis. The fNIRS channels close to the right inferior parietal areas (putative BA 22) also show increased activation to frustrated driving in the HbR t-value maps. Additionally, both HbR and HbO t-value maps show some channels in the left temporo-occipital areas (putative BA 21) to be predictive for frustrated driving although the average linear trend is not as strong there as it is in the frontal areas. Overall, fNIRS revealed brain areas displaying higher activity in the frustrating drives which are in line with the literature on frustration-related neuroimaging lab studies. These areas have been reported to be related with cognitive appraisal, impulse control and emotion regulation processes. Previous research has shown the lateral frontal cortices as a neural correlate for frustration (Siegrist et al., 2005; Hirshfield et al., 2014; Perlman et al., 2014; Yu et al., 2014; Bierzynska et al., 2016). BA 45 and BA 6 are thought to play an important role in modulating emotional response (Olejarczyk, 2007), regulation of negative affect (Ochsner and Gross, 2005; Phillips et al., 2008; Erk et al., 2010), processing emotions (Deppe et al., 2005) and inhibition control (Rubia et al., 2003). BA 22 has been shown to play a crucial role in attributing intention to others (Brunet et al., 2000), and in social perception e.g., processing of non-verbal cues to assess mental states of others (Jou et al., 2010).

The current study has a few limitations that need to be mentioned. First of all, for obtaining the multivariate predictions, the entire Frust condition had been labeled as “frustrated,” while the complete noFrust condition had been labeled as “non-frustrated.” However, it is very likely that the subjectively experienced level of frustration was not constant across the entire drives, because blocking events can temporally increase the level of frustration that also could build up over time (for instance with increasing number of blocking events). We have not considered these factors for our analysis in order to reduce the complexity. In future studies, a more fine-grained analysis of the current frustration level and its development over time could improve the ground truth where the decoding model could discriminate the different levels of frustration (similar to what Unni et al., 2017 achieved for working memory load). Second, stressful cognitive tasks as in the case of frustrated driving may elicit task-related changes in the physiological parameters such as heart rate, respiration rate, blood pressure etc. (Tachtsidis and Scholkmann, 2016). These global components represent a source of noise in the fNIRS data. There are different approaches to monitor these parameters and use them as additional regressors in the GLM e.g., using short-separation fNIRS channels to capture the effects of these physiological signals (Saager and Berger, 2005) or using principle component spatial filtering to separate the global and local components in fNIRS (Zhang et al., 2016). These approaches have been reviewed by Scholkmann et al. (2014). In our study, for the fNIRS analysis, we did not separate the influence of these global components from the intracerebral neural components. However, the localized predictive activation we find renders it unlikely that global physiological effects contribute significantly to our results.

Third, due to the study design with two different participant cohorts, we could not combine the decoding models from the two experiments into one single prediction model. We separated the two experiments because we wanted to have a free view on participants’ face, which is not covered (partly) by the fNIRS cap. Since the results revealed that facial expression of frustration primarily includes activity in the mouth region, we assume that a combination of both measures is feasible, so that future studies should investigate the potential for frustration detection using a combination of facial expressions and brain activity.

Another minor limitation is that we did not use the same subjective questionnaires in the two experiments, so we did not explicitly ask the participants to report the frustration level in the second experiment. Still, the valence and frustration ratings in the first experiment were highly correlated. Moreover, the valence and arousal ratings in were comparable in both experiments and in line with the classification of frustration according to dimensional theories of emotion (Russell, 1980), so that a successful induction of frustration in both experiments is likely.

## Conclusion and Outlook

This study demonstrated the potential of video recordings from the face and whole head fNIRS brain activation measurements for the automated recognition of driver frustration. Although the results of this study are relatively promising, future research is needed to further validate the revealed facial muscle and brain activation patterns. In addition, a combination of both measures (potentially even together with further informative parameters such as peripheral physiology) appears auspicious for improving our models of driver frustration thereby boosting the classification accuracy. The availability of wireless and portable fNIRS devices could make it possible to assess driver frustration *in situ* in real driving in the future. Overall, our results pave the way for an automated recognition of driver frustration for usage in adaptive systems for increasing traffic safety and comfort.

## Author Contributions

KI, AU, JR and MJ planned the research. Data collection was done by KI and AU. Data analysis was carried out by KI, AU, MZ and JR. KI, AU, MZ, JR and MJ prepared the manuscript. KI and AU contributed equally.

## Conflict of Interest Statement

The authors declare that the research was conducted in the absence of any commercial or financial relationships that could be construed as a potential conflict of interest.

## Footnotes

^1^https://www.nitrc.org/projects/fnirs_downstate

^2^http://neurosynth.org

^3^https://www.nitrc.org/projects/mricron

## Supplementary Material

FIGURE S1 Bonferroni-corrected *t*-value maps obtained from channel-wise linear regression of **(A)** HbR and **(B)** HbO fNIRS data for the group-level analyses for the (Frust-noFrust) condition using Generalized Linear Model (GLM). The red and yellow dots represent the sources and detectors, respectively.
