# Supplementary figures and images for: Recognizing Frustration of Drivers From Face Video Recordings and Brain Activation Measurements With Functional Near-Infrared Spectroscopy
Source: Front Hum Neurosci. 2018 Aug 17;12:327. doi: 10.3389/fnhum.2018.00327 (PMC6109683; doi:10.3389/fnhum.2018.00327)

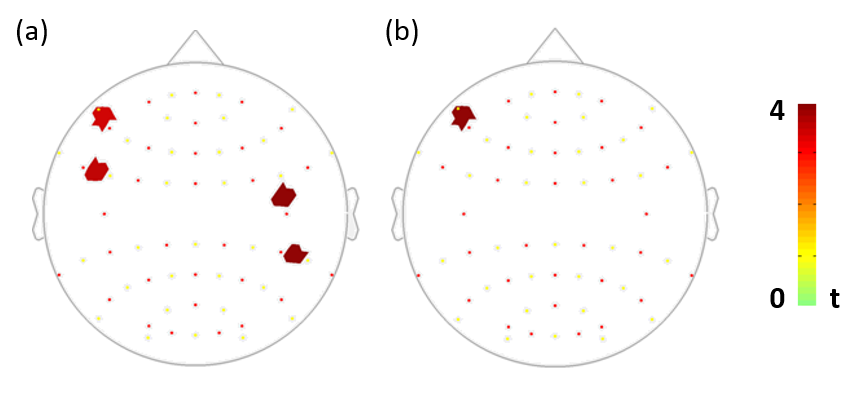

Supplement: FIGURE S1 — Bonferroni-corrected t-value maps obtained from channel-wise linear regression of (A) HbR and (B) HbO fNIRS data for the group-level analyses for the (Frust-noFrust) condition using Generalized Linear Model (GLM). The red and yellow dots represent the sources and detectors, respectively. [file Image_1.TIFF]
